# Measuring recall of medical information in non‐English‐speaking people with cancer: A methodology

**DOI:** 10.1111/hex.12614

**Published:** 2017-09-22

**Authors:** Ruby Lipson‐Smith, Amelia Hyatt, Alexandra Murray, Phyllis Butow, Thomas F. Hack, Michael Jefford, Uldis Ozolins, Sandra Hale, Penelope Schofield

**Affiliations:** ^1^ Cancer Experiences Research Peter MacCallum Cancer Centre Melbourne VIC Australia; ^2^ School of Psychology University of Sydney Sydney NSW Australia; ^3^ Centre of Medical Psychology and Evidence‐Based Decision‐Making University of Sydney Sydney NSW Australia; ^4^ Psycho‐Oncology Cooperative Research Group University of Sydney Sydney NSW Australia; ^5^ College of Nursing University of Manitoba Winnipeg MB Canada; ^6^ CancerCare Manitoba Research Institute Winnipeg MB Canada; ^7^ School of Health Sciences, University of Central Lancashire Preston UK; ^8^ Sir Peter MacCallum Department of Oncology University of Melbourne Melbourne VIC Australia; ^9^ School of Humanities and Languages University of New South Wales Sydney NSW Australia; ^10^ Department of Psychological Sciences Swinburne University of Technology Melbourne VIC Australia

**Keywords:** consultation content, medical information, memory, migrant, non‐english speaking, oncology

## Abstract

**Background:**

Many patients who require an interpreter have difficulty remembering information from their medical consultations. Memory aids such as consultation audio‐recordings may be of benefit to these patients. However, there is no established means of measuring patients’ memory of medical information.

**Objectives:**

This study aimed to develop a method for eliciting and coding recall of medical information in non‐English‐speaking patients.

**Design:**

This method, called Patient‐Interpreter‐Clinician coding (PICcode), was developed in the context of a phase II trial conducted in two outpatient oncology clinics in Melbourne, Australia, and was refined iteratively through consultation with an expert panel and piloting. Between‐coder differences in early versions of the coding system were resolved through discussion and consensus resulting in refinements to PICcode.

**Results:**

The final version of PICcode involved transcribing, translating and coding of audio‐recorded consultations and semi‐structured interviews (SSI). The SSIs were designed to elicit patients’ free‐recall of medical information. Every unit of medical information in the consultation was identified and categorized in a coding tree. SSIs were coded to identify the extent to which information was recalled from the consultation.

**Discussion:**

The iterative changes involved in developing PICcode assisted in clarifying precise details of the process and produced a widely applicable coding system. PICcode is the most comprehensively described method of determining the amount of information that patients who use an interpreter recall from their medical consultations. PICcode can be adapted for English‐speaking patients and other healthcare populations.

## 1 INTRODUCTION

Many patients have difficulty remembering information from their medical consultations, which can negatively impact their understanding of their medical condition, adherence to treatment, management of side‐effects and subsequent outcomes.^1^, ^2^, ^3^, ^4^, ^5^, ^6^ Various interventions have been proposed to help patients remember medical information including written summaries, audio‐recordings, question prompt lists and clinician communication training.^7^

Patients who require an interpreter face additional communication challenges and experience difficulties communicating with their healthcare team.^8^, ^9^ Memory aids may therefore be of particular importance to these patients. Systematic reviews of the literature suggest that providing English‐speaking patients with an audio‐recording of their consultation improves their memory of information provided in the consultation.^10^, ^11^, ^12^, ^13^, ^14^ Two studies have piloted the provision of consultation audio‐recordings to non‐English‐speaking migrant patients.^15^, ^16^ These studies focused on the patients’ perceptions of receiving the audio‐recording and, while both found that patients valued receiving it, neither study measured the impact of the audio‐recording on patients’ memory of information given in the consultation. While subjective perceptions of value are useful, evidence of the effectiveness of communication interventions is needed before recommendations for more widespread implementation can be made.

There are no widely accepted, established means of measuring patients’ memory of medical information, and no detailed methodological account has been published. A variety of studies measuring memory in English‐speaking patients have emerged from diverse areas of health care, including genetic counselling,^17^ surgery,^3^ psychiatry,^5^ gastroenterology,^1^ health behaviour^18^ and oncology.^19^ These studies vary considerably in the type of memory assessed and the measurement methods used. Some measure how much patients remember under usual care conditions and others measure the influence of a new intervention or approach. Most measure what is remembered from a consultation,^20^, ^21^ while others investigate patients’ memory of a therapy,^5^ test results^6^ or health services.^22^ The lack of a consistent measurement tool has hindered efforts in this area. An established measurement process would allow for comparison between interventions, greater consistency between trials and generalizability of results.

Memory retrieval can be broadly categorized into two types: recall and recognition. When evaluating the impact of an intervention, it is important to define the type of memory retrieval being measured as they differ in mechanism and difficulty. Recall involves the retrieval of information from memory without prompting (free recall) or with little prompting (cued recall); while recognition is entirely prompted and involves the identification of a piece of information as either familiar or novel.^23^ It is harder to recall something than to recognize it, and information recalled by patients tends to differ from the information they recognize.^4^, ^24^ A patient's ability to freely recall information is somewhat harder to quantify than their ability to recognize information. Measuring recall requires the researcher to elicit the remembered information from the patient without prompting. The researcher must then identify each item of information provided and determine which ones were recalled. Although harder to measure, the act of recalling information is arguably a more accurate representation of the lived experience of remembering medical information, as patients will often need to remember information without prompting.

In general, there are three steps involved in measuring how much a patient remembers from their consultation: (i) determine the content of the consultation, (ii) determine what the patient remembers from the consultation, and (iii) compare what the patient remembers with the genuine content of the consultation. Many previous studies have used audio‐ or video‐recordings to determine consultation content,^1^, ^4^, ^17^, ^19^, ^20^, ^21^, ^25^, ^26^, ^27^, ^28^, ^29^, ^30^, ^31^ or a combination of recordings and subjective measures such as clinician report.^32^, ^33^ Alternatively, the information delivered has been transcribed verbatim by an observer during the consultation;^3^ or a checklist has been provided to the clinician to ensure that the same information is delivered to each patient.^34^

An audio‐ or video‐recording of a consultation provides the most objective record of the content of the consultation. This recording must then be analysed to label and count the information discussed. Dunn et al^19^adapted the Roter Interaction Analysis System (RIAS)^35^, ^36^ and coded the audio‐recorded content of the consultation into *units of information*, with each unit defined as “a segment of speech from the doctor expressing a single idea concerning medical issues.” Defining units of information using this methodology provides a quantitative summary of consultation content which can then be compared to what the patient remembers from the consultation. Previous studies have varied in how they measured patients’ memory of consultations. Some studies in which the target consultations were highly similar for each patient (eg, chemotherapy education consultations) utilized a standard consultation content questionnaire with multiple choice response options.^27^, ^37^, ^38^ Other studies have used a questionnaire template and personalized this for each patient according to the content of their consultation.^1^, ^17^, ^20^ The multiple choice sections of these questionnaires measure recognition, while other, more open‐ended questions give a measure of cued recall. Both of these approaches limit the consultation types to those where the content is highly similar or consistent between patients. Most studies involving consultations with highly varied content have interviewed the patients post‐consultation.^19^, ^21^, ^26^, ^28^, ^33^, ^39^, ^40^, ^41^

The three steps outlined above also apply to non‐English‐speaking patients; however, further considerations are necessary with this population. Consultations in which interpreters are involved differ from English‐only consultations because all speech needs to be interpreted into another language. Competent, trained interpreters will interpret accurately, but interpretation is an extremely difficult task. There is the risk that nuance of meaning can sometimes be lost, especially when interpreters are not adequately trained.^42^, ^43^, ^44^ The consultation as perceived by a non‐English‐speaking patient is reflective of what is spoken by the interpreter, which may be subtly different from what was spoken by the clinician. However, patients who utilize the services of an interpreter will vary in their ability to understand English and so some may understand parts of what is said in English by the doctor. In such cases, the genuine content of the consultation is less straightforward to determine: Is it reflective of the clinician's input, or the interpreter's interpretation, or a mixture of both? Additionally, determining what the patient remembers requires bilingual research staff, or some other interpretation and translation solution. Any methodology for determining non‐English‐speaking patients’ recall of consultation content must therefore account for these additional variables.

The current study aimed to develop a method for eliciting and coding recall in non‐English‐speaking patients. This method, called Patient‐Interpreter‐Clinician coding (PICcode), involved coding the medical information provided in an oncology consultation where an interpreter was utilized, eliciting the patient's recall of that consultation, and coding the extent of their recall of the information provided in the consultation. This study was conducted in the context of a phase II trial that evaluated the feasibility and acceptability of providing a consultation audio‐recording to non‐English‐speaking patients diagnosed with cancer, the findings of which are in preparation for publication.

## 2 METHODS

**Figure 1:**
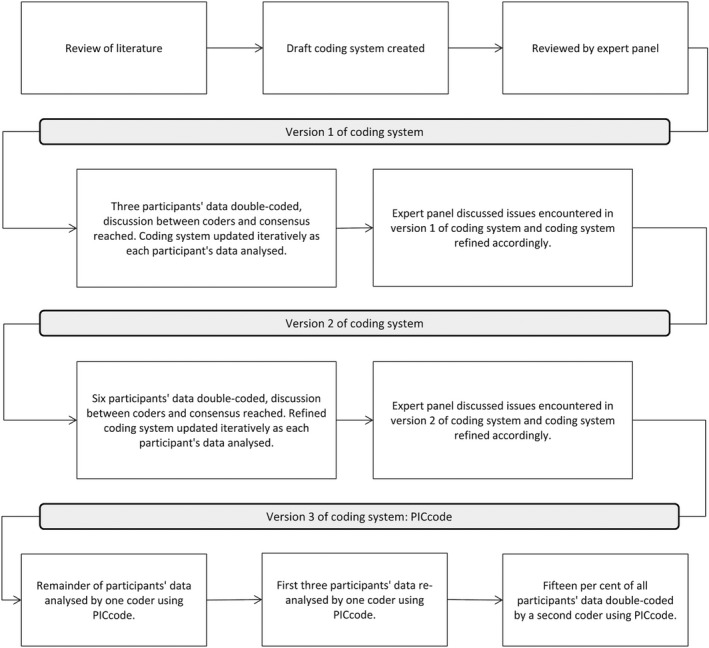
Development of PICcode coding system

The PICcode recall elicitation and coding system was developed iteratively (see Figure 1) using several approaches, including review of existing methods in the literature; piloting; and consultation with experts in consultation audio‐recording, psychology, oncology, coding system development and interpreting/translating (PS, MJ, TH, SH, PB).

### 2.1 Context of development of PICcode

PICcode was developed in the context of a phase II randomized control trial (RCT), which was conducted in two outpatient oncology clinics in Melbourne, Australia. The relevant ethics committees gave approval. Patient eligibility criteria included: aged ≥18 years old; consultation with an oncologist between 1st June 2015 and 1st April 2016; and an Arabic, Cantonese, Greek or Mandarin professional interpreter booked for the consultation. Patients were excluded if they were: participating in a therapeutic clinical trial; too unwell; hearing, vision or speech impaired; self‐identified as non‐literate; or had a diagnosed cognitive or psychological disorder that would preclude participation. All interpreters were professionally accredited and were employed in‐house by the hospital or through a specialist agency. All study information and materials were provided to participants in their own language.

Mid‐treatment review appointments were not included, because a phase I pilot found them to be not as information‐dense as other consultations.^16^ All other consultations with an oncologist were included. Participants had one consultation audio‐recorded using a Dictaphone. The attending interpreter, clinician and patient's family gave prior consent. Audio‐recordings were given to participants on USB or CD. Semi‐structured interviews (SSIs) were used to elicit participants’ recall of the information given in the consultation. Participants completed the SSI via telephone 2 weeks after their audio‐recorded consultation. The SSIs were conducted by bilingual research assistants (RAs). The bilingual RAs were not aware of the content of the audio‐recorded consultation.

### 2.2 Translation of consultations and SSIs

The consultations and SSIs were conducted in English and Arabic, Cantonese, Greek or Mandarin. All data were translated into English prior to analysis so that coding of all languages could be completed by one coder to maintain consistency. Prior to translation, consultation and SSI audio‐recordings were transcribed verbatim by bilingual RAs in the languages in which they were spoken, using the alphabet most common to that language (eg, Mandarin transcribed in simplified Chinese characters, and Cantonese in traditional Chinese characters), and in accordance with published recommendations.^45^ All transcriptions were reviewed by a second bilingual RA to ensure accuracy before being translated into English by professional, tertiary‐trained translators accredited by the National Accreditation Authority for Translators and Interpreters.^46^ The coding process for a particular participant did not commence until both their consultation and SSI had been translated.

### 2.3 Development of PICcode

Relevant literature was reviewed and used to capture existing recall elicitation and coding methods. Searches were not limited to oncology, nor to audio‐recording interventions. General consultation content analysis methods not specific to information recall were also reviewed, such as the RIAS and the Medical Interaction Process System (MIPS).^36^, ^47^

After review of the literature, PICcode adopted the approach taken by Dunn et al^19^ to determine the content of the consultation by coding each unit of information in the recording. This approach was chosen as it can be adapted to many consultation types and be personalized for each participant. Using a standardized format, the information provided by the clinician is dissected into units which can be counted and compared to what the participant remembers post‐consultation.

### 2.4 Development of recall elicitation component of PICcode (semi‐structured interview)

The audio‐recorded consultations had highly varied content and leading questions needed to be avoided to ensure that recall (not recognition) was measured. The interviewers (bilingual RAs) were trained to adapt, respond to the participant, explore new information, re‐focus participants and probe for details to encourage participants to expand on what they recalled. Training included: a session on qualitative interviewing including how to encourage free‐recall and avoid leading questions; listening to example audio‐recordings of well‐conducted and badly conducted SSIs; role‐playing SSIs with another interviewer who spoke their language (so each had the opportunity to “be” the patient); and practicing SSIs in English with a mock‐patient (RLS or AH).

The initial set of SSI questions was developed in conjunction with an oncologist (MJ) and piloted in a phase I study, giving interviewers additional practice.^16^ Patient and staff feedback from the phase I study suggested that the duration of the SSI was taxing and that some questions were potentially leading or not always applicable. In addition, there was concern that participants were not focusing on the consultation that was audio‐recorded and were instead recalling information from other consultations.

The language barrier and time required for transcription and translation prevented supervisors giving real‐time feedback on interview quality, so peer review was used to monitor interviewers’ performance. Each interviewer had at least one of their SSIs reviewed by a peer who assessed their interviewing technique according to criteria developed by supervisors.

### 2.5 Development of recall‐coding component of PICcode

After reviewing the literature, a draft recall‐coding system (version 1) was developed, reviewed by the expert pane, and applied to a pilot sample of 3 participants’ data (see Figure 1). RLS and AH independently double‐coded these participants’ data and then compared their coding. Areas of inconsistency or disagreement were discussed, and the coding system was updated to ensure fidelity of future coding and consistency between coders. Changes were reviewed by the expert panel, and their feedback was used to refine the coding system (version 2).

RLS and AH used the version 2 coding system to double‐code the next 6 participants’ data and updated the coding system where necessary. Following a third panel review, the final coding system (version 3)—named PICcode—was articulated in a detailed manual containing instructions and examples. The iterative updates made to the coding system are discussed in more detail in the results.

An independent coder (AM) then used the PICcode Manual to code the remaining data and to re‐code the first 3 participants’ data. Introduction of an independent coder ensured that the manual and methods were comprehensive, comprehensible and could be applied effectively by other researchers. Fifteen per cent of all participants’ data was then double‐coded by RLS to assess the intercoder reliability of PICcode. Coders were blinded to the content of the SSI when coding the consultation.

### 2.6 Analysis

NVivo (QSR International Pty Ltd., Version 10, 2012) was used to identify and code information in the consultations and SSI transcriptions. Demographic and consultation data were analysed using descriptive statistics. Pearson product‐moment correlation coefficient was used to assess relationships between variables. Statistical analysis was completed using R version 3.3.1 (R Core Team, Vienna, Austria).

The consultations were classified as one of three different “consultation types” on the assumption that some types of consultations would contain more information than others. If the audio‐recorded consultation was the patient's first at the present hospital, it was classified as “first in hospital”. If the patient had visited the hospital before, but this consultation was their first with a particular specialist (eg, their previous consultations had been with a surgical oncologist, while this one was with a radiation oncologist), it was classified as “first with specialist”. The remaining consultations were following an investigation or procedure of some kind and so were classified as “post‐scan, surgery or other work‐up”.

Due to variability in the number of units of information that each participant had to remember, the extent of participants’ information recall is reported as a percentage of the total amount of information given in the consultation. Most previous studies, including Dunn et al,^19^ have compared what the patient remembers to the genuine content of the consultation by calculating the percentage of total information that was recalled by the patient. Percentage of information recalled was calculated for each participant as follows:

$$\text{percentage recalled}=\frac{\text{no. of units of information recalled in the SSI}}{\text{no. of units of information in the consultation}}\times 100$$

Intercoder reliability was assessed by comparing coders’ on of the number of units of information that they identified in the participants’ consultations and the percentage of information that they calculated as recalled. In addition, the content of the units of information identified in the consultation by coder 2 (RLS) were compared to those identified by coder 1 (AM) and were labelled as either “Matching” (unit content identical to coder 1), “Added” (an extra unit identified by coder 2, but not coder 1) or “Omitted” (a unit missed by coder 2, but identified by coder 1).

## 3 RESULTS

### 3.1 Participant and consultation characteristics

Forty‐seven patients consented (9 Arabic, 10 Cantonese, 8 Greek, 20 Mandarin). Six participants withdrew prior to their consultation. One participant's consultation was not audio‐recorded due to technical failure, and 1 participant was lost to follow‐up, leaving 39 participants with complete data (6 Arabic, 8 Cantonese, 6 Greek, 19 Mandarin).

Participant, consultation and SSI characteristics are presented in Table 1. Consultations varied in: type, duration (3 minutes to 90 minutes, mean 23 minutes), the number of units of information discussed (10 to 189) and number of people present (56% of consultations had more than 2 people present).

**Table 1:** Participant, consultation and semi‐structured interview characteristics

	Non‐English‐speaking patients (n = 47)
Age in years, mean (SD, range)	61 (11.7, 31‐79)
Sex, n (%)	
Male	28 (60)
Female	19 (40)
Primary language, n (%)	
Arabic	9 (19)
Cantonese	10 (21)
Greek	8 (17)
Mandarin	20 (43)
Spoken English language skill (self‐reported), n (%)^a^	
None	13 (33)
Basic	13 (33)
Intermediate	12 (31)
Advanced	1 (3)
Cancer Type, n (%)	
Bone & Soft Tissue	5 (11)
Breast	3 (6)
Gynaecological	2 (4)
Haematology	2 (4)
Head & Neck	5 (11)
Lower Gastrointestinal	10 (21)
Lung	7 (15)
Upper Gastrointestinal	4 (9)
Urology	9 (19)
Consultation Type, n (%)^a^	
First in hospital	2 (5)
First with specialist	2 (5)
Post‐scan, surgery or other work‐up	35 (90)
Duration of consultation in minutes, mean (SD, range)^a^	23 (17.13, 3‐90)
First in hospital	58.5 (44.55, 27‐90)
First with specialist	27.5 (17.68, 15‐40)
Post‐scan, surgery or other work‐up	20.86 (13.43, 3‐55)
Number of units of info. in consultation, mean (SD, range)^a^	66 (39.48, 10‐189)
First in hospital	136.5 (74.45, 84‐189)
First with specialist	73.5 (7.78, 68‐79)
Post‐scan, surgery or other work‐up	61.85 (35.45, 10‐154)
Number of health professionals in consultation, n (%)^a^	
1	32 (82)
2	5 (13)
3	2 (5)
Number of family member/s in consultation, n (%)^a^	
0	17 (44)
1	18 (46)
2	4 (10)
Duration of semi‐structured interview in minutes, mean (SD, range)^a^	16 (11.61, 5‐62)

a n = 39 due to withdrawals (n = 6), lost to follow‐up (n = 1) and consultation not audio‐recorded (n = 1).

### 3.2 Recall elicitation component of PICcode (semi‐structured interview)

The final SSI comprised 5 questions and 9 optional prompts (see Table 2). These questions formed a framework for the SSI which the interviewers could expand upon. Overly specific questions (such as, “What did the doctor tell you about your prognosis, and what might happen in the future?”) had been removed, leaving a shorter interview framework with more general questions. The interviewer finished the SSI by providing a summary of the participant's account of the consultation and inviting them to add any additional information (see question 5 in Table 2).

**Table 2:** Semi‐Structured Interview to determine patient recall of consultation content

Questions	Suggested additional prompts (optional)
1. Think back to the appointment you had that was audio‐recorded by our study team 2 weeks ago in the [morning/afternoon] on [date] – do you remember this particular appointment?	Do you remember meeting one of our study team in the waiting room before the appointment? If no, prompt: “Her/his name was [XXXX]”. Did you have any family or friends attend the appointment with you? If doesn't remember, prompt: “I think you came with your [wife/husband/daughter/etc.].” Do you remember the doctor's name or who was the appointment with? If no, prompt: “Her/his name was [Dr XXXX].”
2. Could you tell me what this appointment was about?	Do you remember the main things that the doctor talked to you about? Were there any other things that the doctor talked to you about? Can you tell me about them? In this appointment, did the doctor talk to you about anything specifically? Can you tell me more about that?
3. Did you and the doctor talk about what will happen next?	What do you think this means? What do you think this will involve?
4. Overall, what did you think about this appointment you had with your doctor?	How did it go?
5. [Interviewer provides summary of patients answers to all questions.] Does this summary reflect your thoughts and experiences of that consultation?	

To ensure that participants focused on the consultation that was audio‐recorded, rather than recalling information from other recent consultations, questions were added to the beginning of the SSI to orient the participant to the audio‐recorded consultation (questions 1a‐1c in Table 2). Interviewers corrected participants’ answers to these questions if necessary.

Participants were made aware upon consent that the purpose of the SSI was to talk about their audio‐recorded consultation; but the specific purpose of information recall was only revealed at the time of the SSI so that participants could not actively prepare.

Despite all interviewers receiving equal and comprehensive training, coders’ field notes suggested that interviewers differed in their approach and that the quality of the SSI varied as a result. In some SSIs, the interviewer did not probe for further information when the opportunity arose, or did not ask any questions beyond those in Table 2. The duration of the SSI varied (5 to 62 minutes, mean 16 minutes), and was moderately correlated with both the duration of the consultation (*r *= .50) and the number of units of information in the consultation (*r *= .47). However, coders observed that shorter SSIs were generally not as well conducted.

### 3.3 Recall‐coding component of PICcode (consultation and SSI)

**Figure 2:**
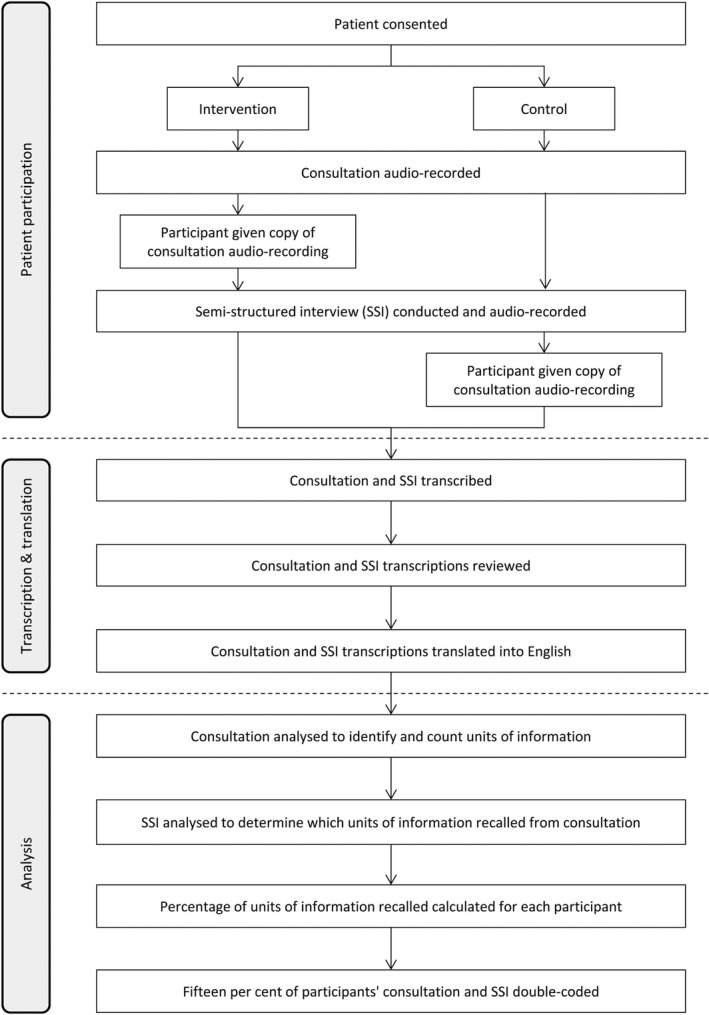
Steps involved in analysing data using PICcode

**Figure 3:**
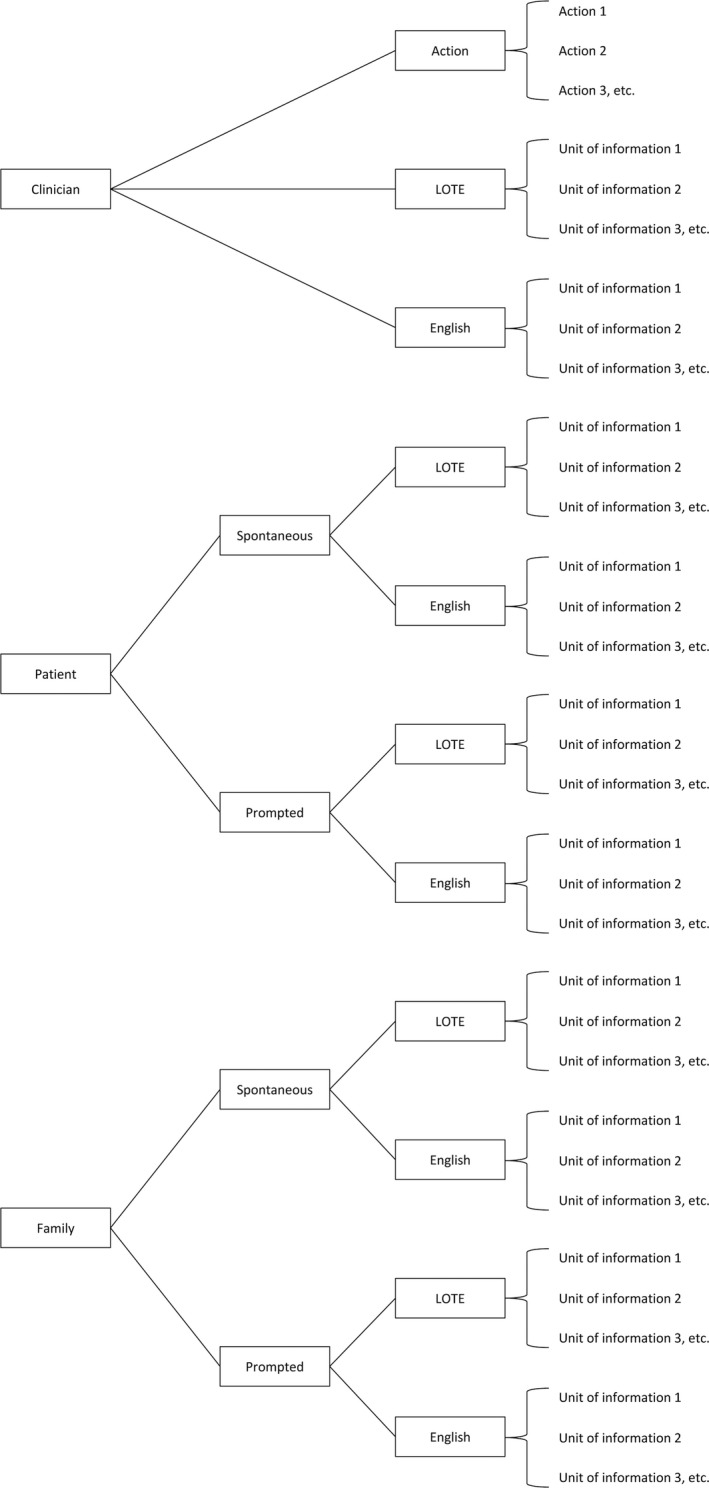
PICcode coding tree

Figure 2 shows the steps in the final PICcode coding system. The PICcode manual provides more detail (supplementary material). Once translated, the transcript of the consultation was read by the coder and every unit of information in the consultation was identified and individually labelled. A unit of information was defined as: a segment of speech expressing a single idea concerning medical issues spoken in a language that could be understood by the patient. The labelled units of information were put into nested categories in a coding tree according to who generated the information, and the language in which it was spoken (see Figure 3). As would be expected, the number of units identified in the consultations was strongly correlated with the duration of the consultations (*r *= .92).

After all units of information from a consultation had been identified and nested in the PICcode coding tree, the SSI transcript was read by the coder to identify what information from the consultation had been recalled by the participant. A unit of information from the consultation was noted as recalled if it was mentioned by the participant in the SSI.

The between‐coder differences that emerged in version 1 and 2 of the coding system prompted inclusion of the following clarifications in PICcode:

1. What to code in the consultation: Using version 1 and 2 of the coding system, the coders occasionally differed in what they considered a *single idea*. For example, the statement “your treatment will cost $5 000‐10 000” could be coded as one unit of information (*treatment will cost $5 000‐10 000*) or as two units of information (*there will be a cost to treatment* and *the cost will be $5 000‐10 000*). The PICcode manual instructs coders to code this sort of layered statement into multiple units of information as the participant could potentially remember one part of the statement (*there will be a cost to my treatment*) without remembering the other part of the statement (*the cost of my treatment will be $5 000‐10 000)*. This allows the extent of a participant's recall to be more accurately represented as the information is deconstructed into separate units that could potentially be remembered independently of each other. The definition of a *medical issue* required clarification. In PICcode, psycho‐social information is considered a medical issue, but general conversation or rapport building is not, unless it contains clinically relevant information.

- Who to code in the consultation: Version 1 of the coding system assumed that only information spoken by the clinician would be coded; however, initial coding attempts suggested that excluding information provided by other parties in the consultation may result in an incomplete account of the consultation and therefore lead to a misrepresentation of the participant's recall ability. Information may be provided in a consultation by the patient, family members or other health professionals. Hence, the PICcode manual instructs that units of information spoken by the patient, family members or other health professionals should be coded. Coding the information generated by the patient in the consultation has the additional benefit of providing a proxy measure of the extent of the patient's involvement in the consultation. The PICcode coding tree distinguishes between clinician‐generated information and patient or family member‐generated information (both prompted and un‐prompted) so that comparisons can be made between the sources of information and the extent of the participant's recall of that information (see Figure 3).
- Which language to code: Version 1 of the coding system assumed that only information spoken in the participant's primary language in the consultation would be coded, as any information spoken in English would not necessarily have been understood by the participant. However, participants’ English language skill varied (see Table 1). At times, a doctor and patient would converse in basic English, and the interpreter would not interpret this exchange. The PICcode coding tree distinguishes information spoken in the participant's first language from information spoken in English where no attempted interpretation was made (see Figure 3). This allows for comparisons to be made between participants’ recall of information spoken in English (that the interpreter had determined was understood by the patient) and information spoken in their primary language.
- What to code as recalled in the SSI: Using version 1 of the coding system, coders occasionally differed in which units of information they considered recalled in the SSI. Coders were therefore instructed to discuss with a colleague if a participant's response in the SSI is ambiguous. If a participant recalls a unit of information that implies recall of another, related, unit of information, then both should be coded as recalled.

### 3.4 Intercoder reliability

After coding was completed, 15% (n = 6; 1 Arabic, 1 Cantonese, 1 Greek, 3 Mandarin) was double‐coded to assess intercoder reliability. Coder discrepancy in the per cent of information recalled by each participant ranged between 0 and 8% (see Table 3). The content of the units of information identified by coder 2 was identical to the content of the majority of the units of information identified by coder 1 for all participants. The per cent of units of information that were identical between coders varied between 63 and 90% (see “Matching” units of information, Table 3). Compared to coder 1, coder 2 identified between 0 and 16 additional units of information for each participant and omitted between 3 and 24 units of information for each participant (see “Added” and “Omitted”, Table 3).

**Table 3:** Comparison between individual coders for 15% of participants

Consultation	Consultation duration (minutes)	Units of information in consultation (n)		Information recalled (%)		
		Coder 1	Coder 2	Coder 1	Coder 2	Coder discrepancy
1	18	65	63 (M = 56, A = 7, O = 9)	25	19	6
2	20	36	32 (M = 28, A = 4, O = 8)	40	38	2
3	14	64	45 (M = 40, A = 5, O = 24)	47	43	4
4	6	24	24 (M = 21, A = 3, O = 3)	42	42	0
5	8	22	16 (M = 16, A = 0, O = 6)	43	44	1
6	16	39	51 (M = 35, A = 16, O = 4)	33	25	8

M, Matching; A, Added; O, Omitted.

## 4 DISCUSSION

This study developed a comprehensive and rigorous methodology for measuring patients’ recall of information from a consultation. PICcode was designed for assessing recall in oncology patients who use the services of an interpreter, but can be adapted for English‐speaking patients and other healthcare populations, making the system relevant and versatile.

The strength of PICcode lies largely in its precision and adaptability. The iterative process involved in developing PICcode assisted in clarifying precise details and produced a coding system that allows for broad applicability. Units of information are coded into a nested coding tree (Figure 3), so that the source and language of the information is specified. This gives PICcode the flexibility to answer a variety of questions about the relationship between the source of information and its later recall. Unlike methods used to measure patients’ recognition of information, PICcode can be applied to different types of consultations with varied content and does not require the consultation content to be predictable.

Existing consultation content analysis systems, such as RIAS and MIPS, have provided processes for breaking the consultation into chunks of information and focus on the quality or content of this information.^48^ Another recent coding system, KINcode, describes how to capture communication and decision‐making behaviours of patients’ family members in the consultation.^49^ PICcode builds on these previous systems to develop an adaptable methodology that can give an indication of patients’ information recall and that is tailored for consultations that require an interpreter. In addition to measuring the impact of interventions designed to improve information recall, PICcode could also be used to assess naturally occurring patient‐driven memory aids, such as taking notes or having family members in the consultation.

Previous information recall studies have defined medical information as information provided by the doctor,^19^ in line with the traditional view of clinician‐led didactic consultations. The contemporary view of a medical consultation describes a triadic interaction between clinician, patient and family.^50^ Shared medical decision‐making has many benefits for both patients and healthcare providers, is valued by many patients and should be the norm for all consultations; it is therefore important to monitor the extent of shared decision‐making so that practices that promote it can be encouraged.^51^, ^52^ Like RIAS, MIPS and KINcode, PICcode acknowledges the triadic nature of the consultation, codes information provided by all parties and could be used to determine patient or family involvement in a consultation.^36^, ^47^, ^49^ The number of units of information provided by the patient or family members can be expressed as a percentage of the total information in the consultation to give an impression of the amount contributed by the patient or family. In addition, distinction can be made between information that the patient or family has spontaneously contributed and prompted information that they provided in response to a question from the clinician. The ratio between spontaneous and prompted information may give an indication of the patient's or family member's sense of agency. Future studies could use PICcode to investigate differences in patients’ memory for medical information delivered by various parties (eg, do patients more easily remember information provided by clinicians than by family?).

While all participants in this study required an interpreter, their English skills varied, with the majority (67%) reporting that they had some English skills. PICcode accounts for patients’ bilingual or multilingual status and can be used in groups with varying English skills to explore the relationship between language skill and information recall. Although PICcode was developed for patients who require an interpreter, it draws on similar research with English‐speaking patients^19^ and can be adapted for use in populations where no interpretation or translation is required. Patients with low‐health literacy are also at risk of not understanding or remembering information from their medical consultations.^53^ PICcode could be adapted for this population and used to investigate the efficacy of interventions designed to improve health literacy.

The consultations that were audio‐recorded and used to develop PICcode were complex and highly varied in content. The number of units of information in a consultation varied between 10 and 189, while consultation duration varied from 3 to 90 minutes, demonstrating that some consultations were very long and information‐dense. Unsurprisingly, consultations that were the patient's first at the present hospital were the longest and most information‐dense. Moreover, some consultations had up to five people present—including doctors, other health professionals, family member/s, the interpreter and the patient. Some clinicians or interpreters are naturally clearer in their communication than others and therefore easier to code; and longer, more complex consultations are difficult to code consistently. Despite this, PICcode was effective in measuring recall of information delivered in the varied, long, complex and information‐dense consultations, and the intercoder reliability was acceptable overall (discrepancy between coders in the per cent of information recalled was between 0 and 8%).

Some limitations were identified in PICcode, primarily in the inconsistency with which the recall elicitation component (SSI) was conducted. Despite the training completed by all the interviewers, the duration of the SSI was only moderately correlated with the amount of information in the consultations, suggesting that there were other factors that influenced the duration, and perhaps quality of the SSIs. The time involved in transcription and translation meant that all SSIs had been conducted before coding began, so coders could not give real‐time feedback to the interviewers. Conduct of the SSIs could be improved in future research by running the interviewing and coding processes in parallel, allowing coders to give feedback to the interviewers. Alternatively, studies involving non‐English‐speaking participants could forego the benefits of a single coder and have bilingual RAs complete both the interviewing and coding, thereby ensuring that interviewers experience the coding process prior to interviewing. Having the interviews and coding completed by bilingual RAs would have the additional benefit of removing the need for translation into English, which is a costly and time‐consuming process. It is recommended that the interviewer training used in the development of PICcode be expanded to include additional scheduled periods of comprehensive interviewer and coder training, frequent refresher training, discussion between coders and more practice trials with simulated patients.

PICcode has been designed to be adaptable. Future studies could expand PICcode to investigate the relationship between patient recall and information salience or importance. Previous studies have compared patients’ recall ability of different types of information by asking clinicians to weight the importance of particular items, or categorizing information according to content (eg, diagnosis, treatment options, prognosis).^1^, ^19^, ^21^ The importance of information could easily be included in the PICcode process by asking clinicians to weight the units of information discussed in the consultation, or by asking patients during the SSI which units of information are most important to them, or by noting whether certain units of information are repeatedly recalled by patients in the SSI. Similarly, units of information could be grouped and nested under new content categories in the PICcode coding tree (eg, diagnosis, treatment options, prognosis).

## 5 CONCLUSIONS

PICcode, and the methods used to develop it, will introduce more consistency in the measurement of patients’ memory of medical information. PICcode can be applied to a wide range of patients, including non‐English‐speaking and low‐health literacy groups, and can be used to measure many factors pertaining to consultation content including information recall and shared decision‐making.

## CONFLICT OF INTEREST

None declared.
