# Supplementary material for: Measuring recall of medical information in non‐English‐speaking people with cancer: A methodology
Source: Health Expect. 2017 Sep 22;21(1):288–99. doi: 10.1111/hex.12614 (PMC5750741; doi:10.1111/hex.12614)
Supplement: Supplementary file 1 [file HEX-21-288-s001.docx]

Patient-Interpreter-Clinician (PICcode) Manual

For Coding of Consultations and Semi-Structured Interviews

****Authorship list to be added after de-identified review process****

This manual should be read in conjunction with the attached publication, “Measuring recall of medical information in non-English speaking people with cancer: A methodology.” Both documents are necessary reading before carrying out the PICcode process.

Table of Contents

[1. Aim of PICcode 3](#_Toc476497903)

[2. Summary of process 3](#_Toc476497904)

[3. Coding content of consultation 3](#_Toc476497905)

[3.1 Coding units of information 3](#_Toc476497906)

[3.1.1. What is a unit of information? 4](#_Toc476497907)

[3.1.2. Which languages to code? 4](#_Toc476497908)

[3.1.3. Who to code? 6](#_Toc476497909)

[3.1.4. Who not to code? 8](#_Toc476497910)

[3.2. Using NVivo 8](#_Toc476497911)

[3.2.1. PICcode coding tree template 9](#_Toc476497912)

[4. Analysing content of Semi-Structured Interview 9](#_Toc476497913)

[4.1. Coding recalled units of Information 9](#_Toc476497914)

[5. Calculating overall information recall for each participant 11](#_Toc476497915)

[6. Fast facts 12](#_Toc476497916)

[7. Coder consensus 13](#_Toc476497917)

[8. Inter-rater reliability 13](#_Toc476497918)

1. Aim of PICcode

The aim of Patient-Interpreter-Clinician coding (PICcode) is to determine how much information is recalled by patients who use an interpreter during their medical consultations.

2. Summary of process

PICcode coding is conducted once both the consultation and semi-structured interview (SSI) have been audio-recorded, transcribed, reviewed, and translated into English.

Analysis should be completed in the following order for each participant:

1. Code content of consultation
2. Code content of SSI
3. Compare the content of the consultation and the SSI to determine information recall

3. Coding content of consultation

The consultation can be coded once a participant's consultation and SSI have both been transcribed and translated into English. You should read through the consultation before you begin, and it may also help to listen to the audio-recording of the consultation while you read. You may only be able to understand the English in the audio-recording, but you will be able to read the translation of the language other than English **(**LOTE**)** as you listen. It can sometimes be helpful to hear the context, tone, and speed of speech when coding the transcription of the consultation.

When coding the consultation, you will be looking out for medical information. We need to quantify this so that we can determine how much of this information the patient then recalls in their SSI.

3.1 Coding units of information

There is often a lot of information discussed in a consultation, some of which is necessary to code and some of which is not. Essentially, we want to ensure that our coding produces an accurate representation of how much information the patient had to retain in their memory after the consultation. It is helpful to think of the information given and discussed in the consultation as ‘units of information’. In this way we are able to quantify how much the patient had to store in their memory.

3.1.1. What is a unit of information?

For our purposes, a unit of information is defined as **a segment of speech expressing a single idea concerning medical issues**.

General chit-chat should not be considered a medical issue, unless it contains important medical/socio-demographic information (as may be the case with some history taking). Psycho-social information should be included as medical information.

When coding, the aim is to try and limit each unit of information to the smallest units that could be remembered by the patient. If you think that part of a segment of speech could be remembered independently by the patient (i.e. separately from surrounding information), then it should be coded as a separate unit of information, even if it was originally expressed as part of a larger sentence or idea. Generally, if the clinician or patient has expressed two ideas in one sentence, then this should be coded as two separate units of information. For example, “You will need to come in on Tuesday to see the nurse and the surgeon” would be coded as three units of information: 1) Patient needs to come in on Tuesday, 2) patient needs to come in to see nurse, 3) patient needs to come in to see surgeon.

However, some sentences may contain two ideas that could not be remembered independently and so should be coded as one unit of information. For example, “The tumours that went away have now come back again” contains two ideas in it (tumours went away and tumours have come back again), but it could only be reasonably remembered as one unit of information by a patient because remembering that the tumours have come back means that you must remember that they originally went away.

3.1.2. Which languages to code?

Most of the information spoken by the health professional in the consultation will be relayed to the patient by the interpreter in the patient’s own language – or, in some cases, the clinician may speak some sentences in the patient’s first language. If the patient has some English-speaking skills, then the clinician and patient may sometimes converse in English, and the interpreter will not attempt to translate this speech if it is obviously not required. Our coded units of information will therefore fall into two language categories:

1. Units of information spoken in the patient’s first language (by interpreter, clinician, patient, or family member)
2. Units of information spoken in English (by clinician, patient, or family member) and no attempted translation made by the interpreter

These two categories should be clearly delineated from each other so that they can be analysed separately if necessary.

If English to LOTE interpretation was attempted by the interpreter, but the resulting interpretation in LOTE was a slightly inaccurate interpretation of the English, code only the interpretation, do not code the English. If a patient happens to remember what the clinician said in English (rather than what was interpreted by the interpreter), then this should be coded as not recalled as we cannot make an assumption that the patient understood the English, but a note will be made. It will probably be quite rare that the interpretation does not match up with what the original English, and also rare that the patient will remember the English version rather than the interpreted version. Indeed, we have not noted a case where the patient remembers the English version rather than the interpreted version.

Just like coding English as a unit of information (see language category 2 above), English should only be coded as recalled if no attempt at translation was made by the interpreter during the consultation. Here, we can make the assumption that the interpreter was correct in their judgement that the patient understood the information spoken in English and thus could accurately remember this as a unit of information.

If a unit of information is spoken in English (i.e., with no attempted interpretation) at one point in the consultation and then is spoken in the LOTE (i.e., interpreted) at another point in the consultation, then code it under the LOTE. Do not double code it. We cannot assume that the patient understood the English, but they have been given a chance to understand the LOTE, so code it under the LOTE. In other words, LOTE trumps English.

3.1.3. Who to code?

There are often many people in a consultation; the clinician, the patient, family members, the interpreter, other health professionals. A consultation is a conversation. It is not just a case of the clinician giving one bit of information after another to the patient.

For example, the clinician may request that the patient give information about symptoms, or the patient may spontaneously give information to the clinician. Some of the information given by the patient (e.g. “I have a sore chest”) may take up a lot of the conversation and will be salient to the patient. Patients may recall these conversations in the SSI, especially if this content forms the majority of the consultation (e.g. the patient may recall, “I told the clinician I have a sore chest”), so we should not assume that the patient will be most likely to recall what the clinician said as opposed to what they themselves said.

If we do not code the units of information given by the patient in the consultation, then this may give a misrepresentation of the amount of information recalled by the patient in the SSI because the patient’s recall of this part of the conversation would not have been noted. Also, coding the amount of information generated by the patient in the consultation gives an indication of the amount that the patient is involved in the consultation. Information spoken by the patient should therefore be included in analysis. Accordingly, our definition of a unit of information can be expanded to the following categories:

1. **A segment of speech expressing a single idea concerning medical issues spoken by the clinician or other health professional.*** (Clinician Generated)
2. **A segment of speech expressing a single idea concerning medical issues spoken by the patient.** (Patient Generated)
3. **A segment of speech expressing a single idea concerning medical issues spoken by a family member.** (Family Generated)

* Not including questions asked by the clinician/health professional. The content of these questions should be captured when we code the patient’s answers.

Clinical actions or activities surmised from the transcription (such as a physical examination or pointing to scan results on a computer screen) should be coded as units of information, even though they may not have been verbally relayed in the consultation. The clinician may have spent several minutes in the consultation visually describing a scan, so as well as remembering the information the clinician spoke about in relation to the scan, a patient may also remember the action; e.g., “the doctor showed me my scan on her computer.” See the ‘Actions’ category in the PICcode coding tree. These were coded separately, as actions do not usually contain information in and of themselves, but may be important in their own right.

In instances where there is more than one health care professional in a consultation – for example, a patient might speak to not only the clinician, but also a pharmacist, a nurse, or even a researcher – all medical information relayed to the patient should be coded. Therefore, any unit of information spoken by a health care professional should be coded as ‘Clinician Generated’. A discussion with any health care professional other than the clinician should also be coded as an ‘Action’. A patient may not consider the individual units of information spoken by a researcher, for example, as equally important as those spoken by the clinician, however they may recall the presence of a researcher. To delineate between units of information spoken by the clinician and units of information spoken by other healthcare professionals, units of information spoken by other health care professionals should be prefixed with an identifying word. For example: “Pharmacist, Px to take warfarin twice a day” or “Research, Px’s having this treatment are more likely to have clotting”.

If the information spoken by a patient is in response to a question from the clinician, then this should be grouped as a ‘Patient Generated’ unit of information, but under a sub-heading that specifies that it was ‘prompted’. If information spoken by a patient is spontaneously given, then it should be coded under a sub-heading that it was ‘spontaneous’.

Coding will clearly delineate between patient and clinician generated information so that comparisons can be made as to whether the audio-recording improves recall of clinician generated information, patient generated information, or both. This allows for flexibility.

3.1.4. Who not to code?

Unsolicited information from the interpreter (if it occurs) is to be excluded from coding as it not regarded as valid medical information if it is not a translation or an attempted translation of information given by another party in the consultation (i.e., spontaneous advice from the interpreter is not a unit of medical information).

3.2. Using NVivo

Make a new NVivo file for each participant that you will be analysing. You can create an NVivo file containing the PICcode coding tree node structure (see section below), and use this as a template for each participant’s file.

Import the participant’s consultation into this new NVivo file and then find, define, and code each unit of information.

Once you have identified a unit of information, code it by highlighting it and dragging it into a node that describes that unit of information. You will need to create a new node if this unit of information has not been mentioned before. Each node represents and contains one unit of information. Nodes should be prefixed by an identifying ‘buzz word’ to make finding the node simpler when coding recalled information in the SSI. For example: “…quite a big possibility it’s cancerous” may be placed in a node called ‘Diagnosis, probable positive cancer diagnosis’ or ‘Diagnosis, probably has cancer’. If a unit of information is repeated during the consultation it should be placed in the appropriate node so that a record is made of how many times this unit of information is repeated. For example: an interpreter may translate, “The biopsy results show cancer cells” at the beginning of a consultation; and then translate, “I’m afraid that we are very certain that you do have cancer because this is what came back in the biopsy” at the end of the consultation. These translations would both be included in a node called something like ‘Diagnosis, positive biopsy’ or ‘Biopsy, Positive biopsy’. Any other units of information related to ‘biopsy’ would also be coded with this identifier, but in a separate node, for example: ‘Biopsy, repeat biopsy after surgery’.

Each participant will have a new file in NVivo and a new list of nodes. The nodes will be unique for each participant because they will have discussed different units of information with their clinician.

Once coding for the consultation is complete, review the list of nodes for ‘node cleaning’. On re-reading there may be some nodes that are similar, or would not be recalled as two separate units of information and can be merged. This is particularly true when coding very long consults, as often the same information is re-visited numerous times. For example: ‘Blood test, must have a blood test the day before chemo each week’ and ‘Blood test, must have a blood test weekly on a Monday before chemo’ could be coded as the one unit of information referenced twice. ‘Node cleaning’ will make coding the information recalled in the SSI much simpler and more accurate.

3.2.1. PICcode coding tree template

You can create a NVivo file containing the PICcode coding tree node structure and use this as a template - see figure 3 in accompanying publication. For each participant, use only what is applicable of this template, not all of the ‘branches’ will apply to every consultation (e.g. some consultations may not have a family member present).

You should also add a ‘Field Notes’ node for each participant in which you can put your own notes about the consultation or the SSI. For example, you may want to comment about the quality of the SSI, or the translation, or any pertinent feedback from the patient about the study.

4. Analysing content of Semi-Structured Interview

Note: do not read the SSI until consultation coding is complete.

4.1. Coding recalled units of Information

Import the SSI into the participant’s NVivo file and match information that has been mentioned by the patient in the SSI with the units of information that you have coded from the consultation. Units of information that were not mentioned in the consultation (i.e., that may have been remembered from a different consultation) are to be ignored.

Sometimes it may appear to be ambiguous as to whether the patient has remembered a unit of information in full. It may look like the patient has half remembered, or slightly misremembered a unit of information. For example, a patient may have remembered the cost of a treatment as being $8-12,000 when it was actually $5-10,000. This patient has remembered that there is a cost to the treatment, but they have misremembered the amount of the cost. Do you score them as having remembered this unit of information or not? If you find yourself asking this question, then it may be because you have included too many ideas in your unit of information in the consultation, so you will need to go back and change it. If the information about the cost in the consultation is coded as two separate units of information (1 = there will be a cost, and 2 = the cost is $5-10,000) then the patient can be scored as having remembered the first unit of information, but not the second. Separating out the single units of information in the consultation allows you to give a more realistic representation of the extent of patient’s recall in the SSI.

If a unit of information is asked about directly in the SSI and not remembered by the patient, then a note should be made of this. For example if the interviewer says “did the clinician tell you about prognosis?” and the patient says “no”, when the clinician had actually told the patient that their cancer was incurable, then a note should be made on the patient’s output. However, keep in mind that you should be confident that the patient understood the interviewer’s question correctly (i.e., was the meaning of ‘prognosis’ explained to them?). If you are not confident about the patient’s understanding of the question then you should mention this in your note.

On occasion, one unit of information recalled in the SSI may apply to two separate nodes. For example: If a patient recalls ‘The PET scan showed the tumour had shrunk’ this could be coded as remembering both ‘PET, had a recent PET scan’ and ‘PET, PET scan showed the tumour had shrunk’. However, in some of these instances it may become apparent that some further ‘Node cleaning’ should be done, as it is unlikely that units of information will be remembered independently of each other and should be merged. There may also be instances where you can assume memory from what is recalled in the SSI. For example: ‘Blood test, have a blood test every Monday’ can be assumed as remembered if the patient recalls ‘Blood test, have a blood test weekly before chemo’ and ‘Chemo, have chemo every Tuesday’. It is clear that the patient has remembered this information and can be assumed that they are aware their blood test is on a Monday. Once again, give the participant the benefit of the doubt if their responses are suggestive of memory.

If a participant is given an incorrect piece of information in a consultation, which was then corrected (e.g., a clinician may have said, “Come and see me in 3 months. No, actually 6 months would be better.”), then these should have been coded as 2 separate units of information in the consultation since the participant may remember one without remembering the other. If the participant then recalls only the correct unit of information in the SSI, both the incorrect and the correct units of information should be labelled as recalled so as not to penalise the participant for not remembering incorrect information. However, if the participant recalls only the incorrect unit of information, then only the incorrect unit should be labelled as recalled.

If a participant’s response is ambiguous, e.g., it is difficult to ascertain exactly what they are referring to, give the participant the benefit of the doubt if possible, but err on the side of caution and discuss with another coder if you are unsure.

5. Calculating overall information recall for each participant

Export the list of nodes for your participant’s consultation from NVivo into Excel by clicking on the ‘list’ option in the ‘external data’ tab. You should now have a list of the nodes in Excel and a count of how many times each of them is mentioned in the consultation and the SSI.

Calculate the percentage of recalled units of information for that consultation as follows:

$$percentage recalled=\frac{no. of units of information recalled in the SSI}{no. of units of information in the consultation} x 100$$

6. Fast facts

This 1-page ‘fast facts’ provides a summary of the PICcode coding manual and can be used as a quick reference when coding.

If you are uncertain whether or how to code something, go back to the **aim of the analysis**, which is to determine how much information is recalled by patients who use an interpreter during their medical consultations, and use this to guide your coding.

A **unit of information** is defined as a segment of speech expressing a single idea concerning medical issues.

- If segments of a sentence/idea could be remembered separately by a patient, then code them as

separate units of information (even if they were originally expressed in one sentence).

- Units of information may be spoken by health professionals (Clinician Generated), patients (Patient

Generated), or family (Family Generated).

English and other languages:

- Nest your nodes under the language in which they are spoken. The purpose of this is to make it clear

which units of information the patient had the best chance of understanding (i.e. which ones were

spoken in their language).

- LOTE trumps English: If a unit of information is spoken in English (i.e. with no attempted interpretation)

at one point in the consultation and then it’s spoken in the LOTE (i.e. interpreted) at another point in the

consultation, then code it under the LOTE. Do not double code it.

7. Coder consensus

The initial few participants’ data should be coded by two coders who then meet to discuss coding and come to a consensus on all content and recall of all nodes (i.e. all units of information). These coders should discuss all facets of coding (what constituted a node of information, whether it was recalled, and how many instances the information was recalled or repeated, etc.), thus the coding process can be refined iteratively over the first few participants.

In order to compare coders’ analyses, firstly, identify which nodes are the same. Then determine if any had similar nodes but different interpretations, or if any had been collapsed or put into different categorisations of information. Also note nodes which were identified by one coder and not the other.

Secondly, review the nodes which had discrepancies between the two coders. Discuss each node to come to a consensus regarding which to keep, which to remove and which to modify.

Finally, identify agreement between information recalled in the SSI. Note disagreements and differences arising from different interpretations of information/nodes, and come to a consensus regarding recall.

8. Inter-rater reliability

Once coding is completed for all participants, 15% should be double coded by a second coder to ensure inter-rater reliability. At a minimum, the coders should be compared to each other on their final figures for percent of information recalled and the content of the units of information identified in the consultation.
